# Predicting South Korean adolescents vulnerable to obesity after the COVID-19 pandemic using categorical boosting and shapley additive explanation values: A population-based cross-sectional survey

**DOI:** 10.3389/fped.2022.955339

**Published:** 2022-09-21

**Authors:** Haewon Byeon

**Affiliations:** ^1^Department of Digital Anti-aging Healthcare (BK21), Graduate School of Inje University, Gimhae, South Korea; ^2^Department of Medical Big Data, College of AI Convergence, Inje University, Gimhae, South Korea

**Keywords:** obesity, COVID-19 pandemic, CatBoost, machine learning, adolescent

## Abstract

**Objective:**

This study identified factors related to adolescent obesity during the COVID-19 pandemic by using machine learning techniques and developed a model for predicting high-risk obesity groups among South Korean adolescents based on the result.

**Materials and methods:**

This study analyzed 50,858 subjects (male: 26,535 subjects, and female: 24,323 subjects) between 12 and 18 years old. Outcome variables were classified into two classes (normal or obesity) based on body mass index (BMI). The explanatory variables included demographic factors, mental health factors, life habit factors, exercise factors, and academic factors. This study developed a model for predicting adolescent obesity by using multiple logistic regressions that corrected all confounding factors to understand the relationship between predictors for South Korean adolescent obesity by inputting the seven variables with the highest Shapley values found in categorical boosting (CatBoost).

**Results:**

In this study, the top seven variables with a high impact on model output (based on SHAP values in CatBoost) were gender, mean sitting hours per day, the number of days of conducting strength training in the past seven days, academic performance, the number of days of drinking soda in the past seven days, the number of days of conducting the moderate-intensity physical activity for 60 min or more per day in the past seven days, and subjective stress perception level.

**Conclusion:**

To prevent obesity in adolescents, it is required to detect adolescents vulnerable to obesity early and conduct monitoring continuously to manage their physical health.

## Introduction

Obesity is an important health problem among adolescents. Adolescent obesity has been increasing worldwide over the past 20 years (1). The prevalence of obesity among adolescents has been steadily increasing from 9.1% in 2016 to 11.1% in 2019 in South Korea as well (2). Since obesity increases not only the size of adipocytes but also the number of adipocytes, obesity in adolescence is highly likely to develop a chronic disease in adulthood (3). In addition, the obesity of adolescents is highly associated with emotional problems such as depression (4).

Meanwhile, SARS-CoV-2, named coronavirus disease 2019 (COVID-19), appeared in December 2019 (5). As COVID-19 spreads rapidly all over the world, the World Health Organization declared a pandemic caused by COVID-19 on March 11, 2020 (5), and the pandemic is still ongoing as of March 2022. As a result, the global population has faced changes in lifestyle due to the COVID-19 pandemic in the world at large, including social distancing. Particularly, home-based classes have become common in South Korea as the classes for adolescents have shifted from face-to-face classes to online classes during the COVID-19 pandemic (6). There is a possibility that health problems such as obesity may appear among adolescents due to the restriction on school attendance. For example, the balanced school meal is replaced by home meals. Nevertheless, only a few studies have evaluated factors for predicting obesity in adolescents under the disaster due to a pandemic infectious disease such as the COVID-19 pandemic.

Meanwhile, machine learning has been widely used in the disease prediction field in recent years (7). Machine learning has several advantages: it can handle multidimensional and multivariate data and it can discover specific patterns in big data.

In particular, categorical boosting (CatBoost) is the most recently developed model among machine learning models based on gradient boosting, and it uses the ordered boosting technique (8). It has been reported that it minimizes model overfitting and has superior predictive performance in predicting categorical variables than XGBoost and LightGBM (8).

This study used 53,534 adolescents who participated in the national survey in South Korea in 2020. This study identified factors related to adolescent obesity during the COVID-19 pandemic by using machine learning techniques and developed a model for predicting high-risk obesity groups among South Korean adolescents based on the result.

## Materials and methods

### Subjects

This study was a secondary data analysis study that analyzed the Korea Youth Risk Behavior Survey (KYRBS) conducted from August 1 to November 30, 2020. The KYRBS is a part of the national chronic disease monitoring system construction plan (9). It is an anonymous online self-report survey jointly conducted by the Ministry of Health and Welfare, the Ministry of Education, and Korea Disease Control and Prevention Agency to understand the current status and level of South Korean adolescents’ health risk behaviors and to evaluate health indicators for promoting adolescents’ health (9). The KYRBS consists of items covering 15 health behavior-associated domains, such as smoking, drinking, obesity, eating habits, and physical activity. The KYRBS divided South Korea into 16 districts (cities and provinces) and then clustered them into 64 city units according to city size. Afterward, this study extracted 800 survey units (400 middle schools and 400 high schools) by using the systematic extraction method as much as the number of samples allocated to each cluster. This study targeted students between seventh grade and twelfth grade across South Korea. The response rate of the 2020 KYRBS was 94.8%. This study analyzed 50,858 subjects (male: 26,535 subjects, and female: 24,323 subjects) out of all subjects (54,948 subjects) of the 2020 KYRBS between 12 and 18 years old after excluding 1,414 subjects who did not respond to height or weight and 2,676 subjects who were underweight (a body mass index below the 5th percentile).

### Variable measurements

Outcome variables were classified into two classes (normal or obesity) based on body mass index (BMI). BMI was calculated by using recently measured height and weight responded by the subject to the first decimal place. BMI was calculated by dividing weight (kg) by squared height (m^2^). This study classified subjects into percentiles according to BMI by gender and age using the 2020 standard growth chart for children and adolescents (10). This study excluded those with a BMI less than the 5th percentile from the analysis to avoid over-interpretation due to the possibility of false-positive in the process of calculating a BMI (11). This study classified adolescents with a BMI of 25 or higher as obese based on the Obesity Treatment Guidelines (12) of the Korean Society for the Study of Obesity.

The explanatory variables included demographic factors, mental health factors, life habit factors, exercise factors, and academic factors. Demographic factors were gender (male or female), grade, area of residence (urban or rural), and subjective household economic level (high, medium, or low). Mental health factors were stress perception rate (high, moderate, or low), the experience of depressive feeling (sadness and hopelessness for more than 2 weeks) in the past 12 months (yes or no), and smartphone overdependence (experienced a severe conflict in friend or social relationships due to excessive smartphone use: yes or no). Life habit factors were smoking in the past 30 days (yes or no), drinking more than 1 shot of soju, beer, or whiskey in the past 30 days (yes or no), the number of days of having breakfast in the past seven days (none, 1–3 days, 4–6 days, or every day), the number of days of eating fruit in the past seven days (none, 1–2 days, or 3 days or more), the number of days of drinking soda in the past seven days (none, 1–2, 3–4, or 5°days or more), the number of days of having fast food in the past seven days (none, 1–2 days, or 3 days or more), mean sleeping hours per day (less than 4, 5, 6, 7, or 8 h or more), and mean sitting hours per day (6 h or less, or 6 h and more). Exercise factors were the number of days of conducting strength training in the past seven days (none, 1–2, 3–4, or 5 days or more), and the number of days of conducting the moderate-intensity physical activity for 60 min or more per day in the past seven days (physical activities that increase the heart rate to 50–70% higher than the maximum heart rate, such as bicycle riding and power walking) (none, 1–2 days, or 3 days or more). An academic factor was grade (high, medium-high, medium, middle-low, or low).

### Categorical boosting

Categorical boosting (CatBoost) is the most recently announced boosting algorithm, and its performance exceeds the performance of existing XGBoost and LightGBM (8). Particularly its unique characteristic is that it is designed to efficiently handle categorical variables while minimizing model overfitting by using an ordered boosting technique. CatBoost can be used without converting categorical variables into numbers. Especially, it improves performance by automatically applying encoding techniques (one-hot encoding, target encoding, mean encoding, and response encoding) suitable for categorical variables. Moreover, since CatBoost optimizes hyperparameters using an internal algorithm without a separate hyperparameter optimization process, it is easier to use compared to other algorithms that require hyperparameter tuning, which is an advantage of this algorithm. While running CatBoost, this study set the number of trees to 100, learning rate to 0.300, regularization’s Lambda to 3, and the limit depth of individual trees to 6.

### Shapley additive explanation

The correct interpretation of machine learning-based predictive model results has always been a subject of interest. In particular, a more complex model has lower interpretability (black box) even though it can improve predictive power. However, the interpretability of a developed machine learning model is very important for health care workers who apply the model results (13). It is essential in terms of helping people gain insights regarding how to advance models and understand the model development process (13).

The tree-based boosting algorithm used in this study can utilize the function of calculating the variable importance based on impurity criteria provided by scikit-learn. At this time, the variable importance is calculated based on the mean decrease in impurity. Therefore, it can evaluate how much each individual variable contributes to improving the predictive performance of the model in the training data. However, it does not reflect the importance of data not included in the training. Moreover, it tends to consider the cardinality of the variable more importantly. It has another limitation that it cannot identify the variable importance of each individual data point and can check the global variable importance of the entire model.

Shapley additive explanation (SHAP), a framework, has been proposed recently to solve this problem (13). This is developed based on game theory, and it is a model-agnostic method (13). The model-agnostic method can explain the specific contribution of each variable to the target data (13). It was used for interpreting the model results of this study especially because its major advantages were contrastive explanation (i.e., identifying the interdependency between individual variables or between an individual variable and the target variable) and variable importance of each data point (i.e., understanding the importance of a local variable).

### Development and validation of a logistic nomogram

This study developed a model for predicting adolescent obesity by using multiple logistic regressions that corrected all confounding factors to understand the relationship between predictors for South Korean adolescent obesity by inputting the seven variables with the highest Shapley values found in CatBoost. In multiple logistic regressions, adjusted odds ratio (aOR) and 95% confidence interval (CI) were presented to identify the independent relationships between predictors and adolescent obesity.

The developed adolescent obesity predictive model visualized the probability of adolescent obesity using a nomogram based on logistic regression so that medical workers could easily interpret the adolescent groups with a high obesity probability. The nomogram refers to a two-dimensional diagram showing the relationship between multiple risk factors to calculate the predictive probability of disease simply and efficiently. It consists of a point line, a risk factor line, a probability line, and a total point line. The point line is placed at the top of the nomogram to derive the score corresponding to the class of each risk factor. The number of risk factor lines was six, corresponding to the number of risk factors for adolescent obesity in this study. The total point line means the sum of the scores of risk factors. The probability line is the final probability of adolescent obesity prediction calculated based on the total score line, and it is placed at the bottom of the nomogram.

The predictive performance of the finally developed adolescent obesity prediction nomogram was evaluated and analyzed using 10-fold cross-validation. F-measure, the area under the curve (AUC), general accuracy, precision, recall, and calibration plots were used as indicators for evaluating predictive performance. All analysis was performed using Python version 3.8.2.^1^

## Results

### General characteristics of subjects according to the prevalence of obesity after the onset of the COVID-19 pandemic

Table 1 shows the results of the chi-square test, which analyzed the differences between obese adolescents and normal-weight adolescents by general characteristics. Among 50,858 subjects, obese adolescents were 9,081 (17.9%). The results of the chi-square test showed that obese adolescents and normal-weight adolescents were significantly (*p* < 0.05) different in gender, grade, subjective household economic level, stress perception level, the experience of depressive feeling, smoking in the past 30 days, drinking in the past 30 days, the number of days of eating fruit in the past seven days, the number of days of drinking soda in the past seven days, the number of days of having fast food in the past seven days, the number of days of conducting the moderate-intensity physical activity for 60 min or more per day in the past seven days, mean sitting hours per day, the number of days of conducting strength training in the past seven days, and academic performance.

**TABLE 1:** General characteristics of subjects by the prevalence of obesity, n (%).

Variable	Obesity		*p*
	No (*n* = 41,777)	Yes (*n* = 9,081)	
Gender			< 0.001
Male	20,009 (75.4)	6,526 (24.6)	
Female	21,768 (89.5)	2,555 (10.5)	
Grade			< 0.001
7th grade	7,530 (85.4)	1,288 (14.6)	
8th grade	7,405 (85.0)	1,307 (15.0)	
9th grade	7,225 (82.9)	1,493 (17.1)	
10th grade	6,867 (81.7)	1,539 (18.3)	
11th grade	6,798 (80.2)	1,676 (19.8)	
12th grade	5,952 (77.0)	1,778 (23.0)	
Area of residence			0.437
Urban	17,949 (82.0)	3,942 (18.0)	
Rural	23,828 (82.3)	5,139 (17.7)	
Subjective household economic level			< 0.001
High	16,379 (83.0)	3,358 (17.0)	
Medium	20,304 (82.7)	4,243 (17.3)	
Low	5,094 (77.5)	1,480 (22.5)	
Stress perception level			0.002
High	13,981 (81.3)	3,213 (18.7)	
Moderate	18,728 (82.7)	3,920 (17.3)	
Low	9,068 (82.3)	1,948 (17.7)	
Experience of depressive feeling			0.005
No	31,226 (81.9)	6,915 (18.1)	
Yes	10,551 (83.0)	2,166 (17.0)	
Experienced a conflict with an acquaintance due to smartphone overdependence			0.725
No	40,106 (82.1)	8,725 (17.9)	
Yes	1,671 (82.4)	356 (17.6)	
Smoking in the past 30 days			< 0.001
No	37,585 (82.5)	7,980 (17.5)	
Yes	4,192 (79.2)	1,101 (20.8)	
Drinking in the past 30 days			< 0.001
No	37,444 (82.5)	7,926 (17.5)	
Yes	4,333 (79.0)	1,155 (21.0)	
Number of days of having breakfast in the past seven days			0.874
0 day (none)	8,722 (82.2)	1,886 (17.8)	
1–3 days	10,548 (81.9)	2,330 (18.1)	
4–6 days	10,533 (82.2)	2,284 (17.8)	
7 days (everyday)	11,974 (82.3)	2,581 (17.7)	
Number of days of eating fruit in the past seven days			< 0.001
None	5,219 (79.7)	1,331 (20.3)	
1–2 days	24,460 (81.7)	5,469 (18.3)	
3 days or more	12,098 (84.1)	2,281 (15.9)	
Number of days of drinking soda in the past seven days			< 0.001
0 day (none)	9,475 (83.6)	1,858 (16.4)	
1–2 days	17,623 (81.9)	3,893 (18.1)	
3–4 days	9,105 (81.0)	2,139 (19.0)	
5 days or more	5,574 (82.4)	1,191 (17.6)	
Number of days of having fast food in the past seven days			0.009
None	7,484 (81.5)	1,700 (18.5)	
1–2 days	23,838 (82.0)	5,238 (18.0)	
3 days or more	10,455 (83.0)	2,143 (17.0)	
Mean sleeping hours per day			0.945
Less than 4 h	5,173 (82.2)	1,118 (17.8)	
5 h	7,402 (82.0)	1,621 (18.0)	
6 h	8,613 (81.8)	1,814 (18.2)	
7 h	7,081 (81.8)	1,579 (18.2)	
8 h or more	7,523 (82.1)	1,645 (17.9)	
The number of days of conducting the moderate-intensity physical activity for 60 min or more per day in the past seven days			< 0.001
None	16,169 (83.8)	3,136 (16.2)	
1–2 days	12,225 (82.2)	2,652 (17.8)	
3 days or more	10,731 (79.9)	2,707 (20.1)	
Mean sitting hours per day			< 0.001
Less than 6 h	35,491 (82.4)	7,565 (17.6)	
6 h or more	5,502 (80.6)	1,326 (19.4)	
The number of days of conducting strength training in the past seven days			< 0.001
None	20,947 (82.9)	4,316 (17.1)	
1–2 days	10,320 (80.7)	2,462 (19.3)	
3–4 days	5,180 (81.2)	1,200 (18.8)	
5 days or more	5,330 (82.9)	1,103 (17.1)	
Academic performance			< 0.001
High	5,293 (84.9)	941 (15.1)	
Medium-high	10,549 (84.4)	1,948 (15.6)	
Medium	12,743 (82.5)	2,694 (17.5)	
Medium-low	9,301 (79.4)	2,417 (20.6)	
Low	3,891 (78.3)	1,081 (21.7)	

### Predictors for obesity in South Korean adolescents

**FIGURE 1:**
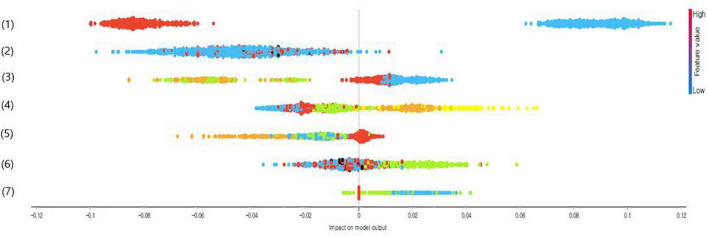
Impact on model output of adolescent obesity predictors using categorical boosting (CatBoost’s) shapley additive explanation (SHAP) values (only top seven variables are presented); (1) Gender (1 = male or 2 = female), (2) mean sitting hours per day (1 = less than 6 h or 2 = 6 h or more), (3) number of days of conducting strength training in the past seven days (1 = none, 2 = 1–2 days, 3 = 3–4 days, or 4 = 5 days or more), (4) academic performance (1 = high, 2 = medium-high, 3 = medium, 4 = medium-low, or 5 = low), (5) the number of days of drinking soda in the past seven days (1 = none, 2 = 1–2 days, 3 = 3–4 days, or 4 = 5 days or more), (6) the number of days of conducting the moderate-intensity physical activity for 60 min or more per day in the past seven days (1 = none, 2 = 1–2 days, or 3 = 3 days, and more), and (7) stress perception level (1 = high, 2 = moderate, or 3 = low).

Figure 1 presents the calculated SHAP value of a factor related to obesity in South Korean adolescents by using CatBoost. In this study, the top seven variables with a high impact on model output (based on SHAP values) were gender, mean sitting hours per day, the number of days of conducting strength training in the past seven days, academic performance, the number of days of drinking soda in the past seven days, the number of days of conducting the moderate-intensity physical activity for 60 min or more per day in the past seven days, and subjective stress perception level.

Table 2 shows the results of logistic regression analysis for predicting obesity in South Korean adolescents using the top seven variables with high impact on model output in CatBoost. The results of the adjusted model for predicting suicidal ideation in South Korean adolescents revealed that independent influence factors were male (AOR = 3.39, 95% CI: 3.20, 3.58), stress perception level moderate or higher (moderate: AOR = 1.08, high: AOR = 1.33), the number of days of drinking soda in the past seven days (1–2 days: AOR = 1.25, 3–4 days: AOR = 1.28, and 5 days or more: AOR = 1.21), the number of days of conducting the moderate-intensity physical activity for 60 min or more per day in the past seven days (1–2 days: AOR = 1.06 and none: AOR = 1.21), number of days of conducting strength training in the past seven days (3–4 days: AOR = 1.25, 1–2 days: AOR = 1.55, and none: AOR = 1.81), and academic performance (medium-high: AOR = 1.12, medium: AOR = 1.33, medium-low: AOR = 1.61, and low: AOR = 1.65) (*p* < 0.05).

**TABLE 2:** Predictors for obesity in South Korean adolescents: aOR and 95% CI.

Variable	AOR	95% CI	*p*
**Gender**			
Male	3.39	3.20, 3.58	< 0.001
Female (reference)	1	1	
**Stress perception level**			
High	1.33	1.25, 1.43	< 0.001
Moderate	1.08	1.01, 1.15	0.015
Low (reference)	1	1	
**Number of days of drinking soda in the past seven days**			
0 day (reference)	1	1	
1–2 days	1.25	1.15, 1.37	< 0.001
3–4 days	1.28	1.18, 1.38	< 0.001
5 days or more	1.21	1.11, 1.31	< 0.001
**The number of days of conducting the moderate-intensity physical activity for 60 min or more per day in the past seven days**			
None	1.21	1.14, 1.29	< 0.001
1–2 days	1.06	1.01, 1,12	0.045
3 days or more (reference)	1	1	
**Mean sitting hours per day**			
Less than 6 h (reference)	1	1	
6 h or more	1.08	1.01, 1.16	0.016
**The number of days of conducting strength training in the past seven days**			
None	1.81	1.65, 1.99	< 0.001
1–2 days	1.55	1.41, 1.70	< 0.001
3–4 days	1.25	1.13, 1.39	< 0.001
5 days or more (reference)	1	1	
**Academic performance**			
High (reference)	1	1	
Medium-high	1.12	1.02, 1.23	0.011
Medium	1.33	1.22, 1.45	< 0.001
Medium-low	1.61	1.47, 1.76	< 0.001
Low	1.65	1.48, 1.83	< 0.001

### Development and validation of a nomogram for predicting groups vulnerable to obesity in South Korean adolescents

**FIGURE 2:**
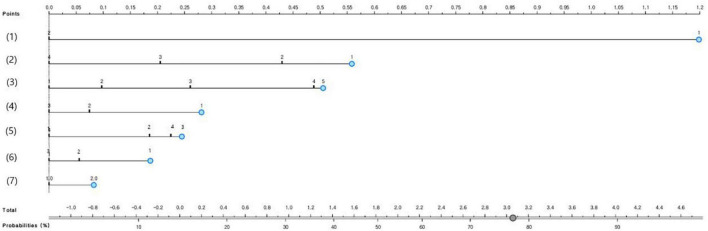
The nomogram to predict groups vulnerable to obesity in South Korean adolescents; (1) Gender (1 = male or 2 = female), (2) number of days of conducting strength training in the past seven days (1 = none, 2 = 1–2 days, 3 = 3–4 days, or 4 = 5 days or more), (3) academic performance (1 = high, 2 = medium-high, 3 = medium, 4 = medium-low), (4) stress perception level (1 = high, 2 = moderate, or 3 = low), (5) the number of days of drinking soda in the past seven days (1 = none, 2 = 1–2 days, 3 = 3–4 days, or 4 = 5 days or more), (6) the number of days of conducting the moderate-intensity physical activity for 60 min or more per day in the past seven days (1 = none, 2 = 1–2 days, or 3 = 3 days and more), and (7) mean sitting hours per day (1 = less than 6 h or 2 = 6 h or more).

Figure 2 presents the nomogram for predicting groups vulnerable to obesity among South Korean adolescents. The nomogram predicted that the probability of male students who currently felt a lot of stress, had poor academic performance, drank soda for 3–4 days in the past seven days, did not conduct the moderate-intensity physical activity for 60 min or more per day or strength training, and sat for 6 h or more per day on average having obesity was 77%.

**FIGURE 3:**
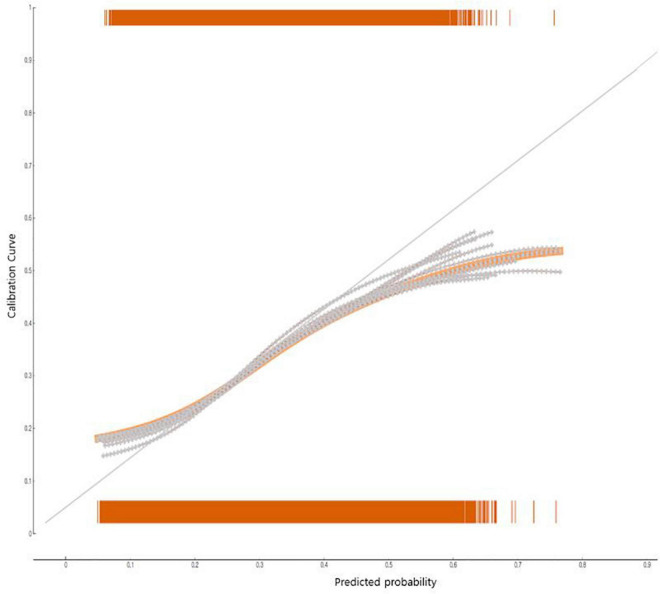
The performance of the nomogram to predict groups vulnerable to obesity in South Korean adolescents: calibration plot.

The developed nomogram for predicting adolescent obesity examined its predictive performance using AUC, general accuracy, F1, recall, precision, and calibration plot (Figure 3). When the predicted probability and the observed probability were compared by using calibration plots and a chi-square test for the obese group and the non-obese group (Figure 3), the predicted probability and the observed probability were not significantly different (*p* < 0.05). The results of 10-fold cross validation showed that the AUC of the adolescent suicidal ideation prediction nomogram was 0.68, the general accuracy of it was 0.82, the precision of it was 0.77, the recall of it was 0.82, and the F-[Frame1] measure of it was 0.78.

## Discussion

This study identified influencing factors associated with obesity in adolescents after the COVID-19 pandemic. The results of this study showed that the obesity rate of adolescents was 17.9%. It was a 2.5% increase from the 2019 national survey (15.4%). Studies in other countries, such as the United States (14) and China (15), also reported an increase in adolescent obesity rates after the declaration of the COVID-19 pandemic. Ministry of Education, Ministry of Health and Welfare, Korea Disease Control, and Prevention Agency (14) showed that the prevalence of obesity among U.S. adolescents increased by more than 15% during the COVID-19 pandemic due to school closures. The prevalence of obesity among Chinese adolescents between 15 and 17 years also increased from 10.5 to 12.9%, more than 2.4% (15). Stavridou et al. (16) systematically reviewed 15 studies (17,028,111 subjects in total) related to adolescent obesity during the COVID-19 pandemic and reported that the obesity rate increased because adolescents had less physical activities as outdoor activities were restricted due to lockdown.

The WHO declared a pandemic on March 11, 2020, as COVID-19 began to spread globally from January 2020 (5). South Korea also implemented intensive social distancing such as shifting schooling from in-person to online classes and restricting indoor and outdoor physical activities to prevent the spread of the infectious disease (17). It has been reported that adolescent obesity is affected by various factors including social and environmental factors and lifestyle factors as well as biological factors. It is believed that the extended COVID-19 pandemic has induced changes in lifestyles such as restrictions on outdoor activities to affect the physical health of adolescents such as obesity as well as their mental health.

In this study, gender was a major predictor of adolescent obesity. The obesity rate of South Korean male adolescents and that of South Korean female adolescents were similar until 2010, but the former tended to be higher than the latter after 2011 (9). In particular, this epidemiologic study examined the obesity rate of South Korean adolescents after the COVID-19 pandemic and showed that male students (24.6%) had a 2.5 times higher obesity rate than female students (10.5%). Therefore, it is necessary to develop obesity management guidelines considering the characteristics of gender to prevent adolescent obesity.

In this study, soda intake was a major risk factor for adolescent obesity. The social distancing restrictions after COVID-19 changed dietary life such as an increase in food delivery and instant food intake (18–21). Four out of 10 Palestinian adolescents gained weight due to increased consumption of soda, fried dishes, and sweets (18). Ing and Ing (19) and Bertens et al. (20) also showed that Italy, Spain, Chile, Colombia, and Brazil adolescents consumed soda and snacks up to 20.7% more than usual during the lockdown period. Particularly, obese adolescents with a high BMI consumed a lot of soda. Absorption of excessive sugar increases lipid synthesis in the liver, which increases the risk of cardiovascular disease and obesity (22). A large-scale epidemiologic study is required to determine the causal relationship between increased soda consumption and adolescent obesity during the COVID-19 pandemic.

Another finding of this study was that a decrease in moderate-intensity physical activity and strength training and an increase in sitting hours were significantly associated with adolescent obesity after the COVID-19 pandemic. Fernandez-Rio et al. (23) revealed that the weight gain of adolescents after the COVID-19 pandemic was significantly related with a decrease in moderate-intensity or higher physical activity, which agreed with the results of this study. In particular, intensive social distancing was implemented in South Korea during the COVID-19 pandemic (24). For example, the business of fitness centers and group sports facilities was restricted during the period (24).

Moreover, Dunton et al. (25) reported that sedentary behaviors increased while the physical activity of adolescents decreased during the lockdown period. Previous studies (26, 27) revealed that screen-based sedentary behavior such as watching TV was closely related to obesity in children and adolescents. Especially, Kwak and Ickovics (27) showed that maintaining a sedentary behavior (e.g., watching TV) for more than 2 h a day also increased the risk of type 2 diabetes, in addition to obesity. Kang et al. (28) on South Korean adolescents also reported that adolescents who spent 35 h or more per week in screen-based sedentary behavior had a higher chance of metabolic disease morbidity than those who spent 16 h or less per week.

Since lockdown included not only indoor social distancing but also restrictions on outdoor activities such as sports, the decrease in strength training or the change in physical activity habits during the COVID-19 pandemic was likely to act as major influencing factors for adolescent obesity. Sekulic et al. (29) showed that a decrease in the physical activity level due to social distancing negatively influenced basic physical strength. In particular, Sekulic et al. (29) observed reduced stamina among male students in relation to their participation in organized sports. As non-face-to-face activities have increased during the COVID-19 pandemic, adolescents had fewer physical activities. As a result, to prevent adolescent obesity, it is necessary to prepare a physical activity promotion program for adolescents vulnerable to obesity.

This study developed a model for predicting adolescent obesity after the COVID-19 pandemic while considering multiple health risk factors. The model showed that the probability of male students who currently felt a lot of stress, had poor academic performance, drank soda for 3–4 days in the past seven days, did not conduct the moderate-intensity physical activity for 60 min or more per day or strength training, and sat for 6 h or more per day on average having obesity was 77%, which was very high. Previous studies showed that an increase in sedentary hours and a decrease in physical activity adversely influenced the stamina and sense of balance of adolescents (30) and decreased physical activities induced stress and obesity in adolescents (31, 32). These results implied the risk of adolescent obesity due to multiple health behaviors. However, it is difficult to compare these results with the results of this study directly. Obese subjects are vulnerable to infectious diseases, and obesity is a major influencing factor for chronic diseases (33). Therefore, to promote the health of adolescents, it is required to develop customized health promotion programs considering the multiple health risk behaviors of adolescents vulnerable to obesity and conduct monitoring continuously at the school level (34). Furthermore, since only a few studies analyzed multiple health risk factors of adolescent obesity, additional epidemiological studies are needed to analyze multiple health risk factors in obese adolescents considering various factors.

The limitations of this study are as follows. First, since this study used an online survey as a data source, this study could underestimate the percentage of obesity. A large-scale epidemiological study is needed to measure adolescent obesity more accurately by using body measurements such as height, weight, and waist circumference. Second, there can be other potential explanatory variables, in addition to the explanatory variables included in this study, that may affect obesity after the COVID-19 pandemic. It is required to develop models for predicting adolescent obesity while including various potential variables that are likely to influence obesity, such as adolescent mental health. Third, the raw data of this study did not have information whether obese adolescents were obese before the COVID-19 pandemic. Therefore, it is difficult to interpret that the obesity observed in this epidemiological study occurred after the COVID-19 pandemic. A retrospective cohort study comparing obesity before and after the COVID-19 pandemic is needed in the future. Fourth, since it was a cross-sectional study, the results of this study could not be interpreted as a cause-effect relationship. Additional cohort studies are needed to determine causality between factors.

## Conclusion

This epidemiological study predicted that male students who currently felt a lot of stress, had poor academic performance, drank soda for 3–4 days in the past seven days, did not conduct the moderate-intensity physical activity for 60 min or more per day or strength training, and sat for 6 h or more per day on average were vulnerable to obesity. Consequently, to prevent obesity in adolescents, it is required to detect adolescents vulnerable to obesity early and conduct monitoring continuously to manage their physical health. Furthermore, schools need to prepare a customized support system that considers the characteristics of multiple health risk behaviors in the high-obesity-risk group.

## Data availability statement

The raw data supporting the conclusions of this article will be made available by the authors, without undue reservation.

## Ethics statement

The studies involving human participants were reviewed and approved by Korea Disease Control and Prevention Agency (protocol code 117075 and date: 2021.07.01). Written informed consent to participate in this study was provided by the participants’ legal guardian/next of kin.

## Author contributions

The author confirms being the sole contributor of this work and has approved it for publication.

## Footnotes

1
https://www.python.org
