# Supplementary material for: Predicting South Korean adolescents vulnerable to obesity after the COVID-19 pandemic using categorical boosting and shapley additive explanation values: A population-based cross-sectional survey
Source: Front Pediatr. 2022 Sep 21;10:955339. doi: 10.3389/fped.2022.955339 (PMC9532523; doi:10.3389/fped.2022.955339)
Supplement: Supplementary file 1 [file Data_Sheet_1.docx]

Supplementary Material

# Supplementary Tables

**Table 1**. General characteristics of subjects by the prevalence of obesity, n (%)

| **Variable** | **Obesity** | | **p** |
| --- | --- | --- | --- |
|  | **No (n=41,777)** | **Yes (n=9,081)** |  |
| Gender |  |  | <0.001 |
| Male | 20,009 (75.4) | 6,526 (24.6) |  |
| Female | 21,768 (89.5) | 2,555 (10.5) |  |
| Grade |  |  | <0.001 |
| 7th grade | 7,530 (85.4) | 1,288 (14.6) |  |
| 8th grade | 7,405 (85.0) | 1,307 (15.0) |  |
| 9th grade | 7,225 (82.9) | 1,493 (17.1) |  |
| 10th grade | 6,867 (81.7) | 1,539 (18.3) |  |
| 11th grade | 6,798 (80.2) | 1,676 (19.8) |  |
| 12th grade | 5,952 (77.0) | 1,778 (23.0) |  |
| Area of Residence |  |  | 0.437 |
| Urban | 17,949 (82.0) | 3,942 (18.0) |  |
| Rural | 23,828 (82.3) | 5,139 (17.7) |  |
| Subjective Household Economic Level |  |  | <0.001 |
| High | 16,379 (83.0) | 3,358 (17.0) |  |
| Medium | 20,304 (82.7) | 4,243 (17.3) |  |
| Low | 5,094 (77.5) | 1,480 (22.5) |  |
| Stress Perception Level |  |  | 0.002 |
| High | 13,981 (81.3) | 3,213 (18.7) |  |
| Moderate | 18,728 (82.7) | 3,920 (17.3) |  |
| Low | 9,068 (82.3) | 1,948 (17.7) |  |
| Experience of depressive feeling |  |  | 0.005 |
| No | 31,226 (81.9) | 6,915 (18.1) |  |
| Yes | 10,551 (83.0) | 2,166 (17.0) |  |
| Experienced a conflict with an acquaintance due to smartphone overdependence |  |  | 0.725 |
| No | 40,106 (82.1) | 8,725 (17.9) |  |
| Yes | 1,671 (82.4) | 356 (17.6) |  |
| Smoking in the past 30 days |  |  | <0.001 |
| No | 37,585 (82.5) | 7,980 (17.5) |  |
| Yes | 4,192 (79.2) | 1,101 (20.8) |  |
| Drinking in the past 30 days |  |  | <0.001 |
| No | 37,444 (82.5) | 7,926 (17.5) |  |
| Yes | 4,333 (79.0) | 1,155 (21.0) |  |
| Number of days of having breakfast in the past seven days |  |  | 0.874 |
| 0 day (none) | 8,722 (82.2) | 1,886 (17.8) |  |
| 1-3 days | 10,548 (81.9) | 2,330 (18.1) |  |
| 4-6 days | 10,533 (82.2) | 2,284 (17.8) |  |
| 7 days (everyday) | 11,974 (82.3) | 2,581 (17.7) |  |
| Number of days of eating fruit in the past seven days |  |  | <0.001 |
| None | 5,219 (79.7) | 1,331 (20.3) |  |
| 1-2 days | 24,460 (81.7) | 5,469 (18.3) |  |
| 3 days or more | 12,098 (84.1) | 2,281 (15.9) |  |
| Number of days of drinking soda in the past seven days |  |  | <0.001 |
| 0 day (none) | 9,475 (83.6) | 1,858 (16.4) |  |
| 1-2 days | 17,623 (81.9) | 3,893 (18.1) |  |
| 3-4 days | 9,105 (81.0) | 2,139 (19.0) |  |
| 5 days or more | 5,574 (82.4) | 1,191 (17.6) |  |
| Number of days of having fast food in the past seven days |  |  | 0.009 |
| None | 7,484 (81.5) | 1,700 (18.5) |  |
| 1-2 days | 23,838 (82.0) | 5,238 (18.0) |  |
| 3 days or more | 10,455 (83.0) | 2,143 (17.0) |  |
| Mean sleeping hours per day |  |  | 0.945 |
| Less than 4 hours | 5,173 (82.2) | 1,118 (17.8) |  |
| 5 hours | 7,402 (82.0) | 1,621 (18.0) |  |
| 6 hours | 8,613 (81.8) | 1,814 (18.2) |  |
| 7 hours | 7,081 (81.8) | 1,579 (18.2) |  |
| 8 hours or more | 7,523 (82.1) | 1,645 (17.9) |  |
| The number of days of conducting the moderate-intensity physical activity for 60 minutes or more per day in the past seven days |  |  | <0.001 |
| None | 16,169 (83.8) | 3,136 (16.2) |  |
| 1-2 days | 12,225 (82.2) | 2,652 (17.8) |  |
| 3 days or more | 10,731 (79.9) | 2,707 (20.1) |  |
| Mean sitting hours per day |  |  | <0.001 |
| Less than 6 hours | 35,491 (82.4) | 7,565 (17.6) |  |
| 6 hours or more | 5,502 (80.6) | 1,326 (19.4) |  |
| The number of days of conducting strength training in the past seven days |  |  | <0.001 |
| None | 20,947 (82.9) | 4,316 (17.1) |  |
| 1-2 days | 10,320 (80.7) | 2,462 (19.3) |  |
| 3-4 days | 5,180 (81.2) | 1,200 (18.8) |  |
| 5 days or more | 5,330 (82.9) | 1,103 (17.1) |  |
| Academic performance |  |  | <0.001 |
| High | 5,293 (84.9) | 941 (15.1) |  |
| Medium-high | 10,549 (84.4) | 1,948 (15.6) |  |
| Medium | 12,743 (82.5) | 2,694 (17.5) |  |
| Medium-low | 9,301 (79.4) | 2,417 (20.6) |  |
| Low | 3,891 (78.3) | 1,081 (21.7) |  |

**Table 2.** Predictors for obesity in South Korean adolescents: aOR and 95% CI

| **Variable** | **AOR** | **95%CI** | **p** |
| --- | --- | --- | --- |
| Gender |  |  |  |
| Male | 3.39 | 3.20, 3.58 | <0.001 |
| Female (reference) | 1 | 1 |  |
| Stress Perception Level |  |  |  |
| High | 1.33 | 1.25, 1.43 | <0.001 |
| Moderate | 1.08 | 1.01, 1.15 | 0.015 |
| Low (reference) | 1 | 1 |  |
| Number of days of drinking soda in the past seven days |  |  |  |
| 0 day (reference) | 1 | 1 |  |
| 1-2 days | 1.25 | 1.15, 1.37 | <0.001 |
| 3-4 days | 1.28 | 1.18, 1.38 | <0.001 |
| 5 days or more | 1.21 | 1.11, 1.31 | <0.001 |
| The number of days of conducting the moderate-intensity physical activity for 60 minutes or more per day in the past seven days |  |  |  |
| None | 1.21 | 1.14, 1.29 | <0.001 |
| 1-2 days | 1.06 | 1.01, 1,12 | 0.045 |
| 3 days or more (reference) | 1 | 1 |  |
| Mean sitting hours per day |  |  |  |
| Less than 6 hours (reference) | 1 | 1 |  |
| 6 hours or more | 1.08 | 1.01, 1.16 | 0.016 |
| The number of days of conducting strength training in the past seven days |  |  |  |
| None | 1.81 | 1.65, 1.99 | <0.001 |
| 1-2 days | 1.55 | 1.41, 1.70 | <0.001 |
| 3-4 days | 1.25 | 1.13, 1.39 | <0.001 |
| 5 days or more (reference) | 1 | 1 |  |
| Academic performance |  |  |  |
| High (reference) | 1 | 1 |  |
| Medium-high | 1.12 | 1.02, 1.23 | 0.011 |
| Medium | 1.33 | 1.22, 1.45 | <0.001 |
| Medium-low | 1.61 | 1.47, 1.76 | <0.001 |
| Low | 1.65 | 1.48, 1.83 | <0.001 |
